# Intravenous thrombolysis before percutaneous coronary intervention in patients with non-ST-elevation acute coronary syndrome and acute ischaemic stroke: a subanalysis of the PRAISE study

**DOI:** 10.1136/openhrt-2025-003567

**Published:** 2025-09-29

**Authors:** Annahita Sedghi, Regina von Rennenberg, Gabor Petzold, Georg Nickenig, Bernd Kallmünzer, Stephan Achenbach, Roman Huber, Julia Seeger, Bettina von Sarnowski, Goetz Thomalla, Peter Arthur Ringleb, Dominik Michalski, Ulrich Laufs, Georg Royl, Kristina Szabo, Norman Mangner, Volker Puetz, Lars Kellert, Stefan Kaeaeb, Silke Wunderlich, Karl-Ludwig Laugwitz, Martina Petersen, Annerose Mengel, David M Leistner, Ulf Landmesser, Matthias Endres, Christian H Nolte, Timo Siepmann

**Affiliations:** 1 Department of Neurology, Medical Faculty and University Hospital Carl Gustav Carus, TUD Dresden University of Technology, Dresden, Germany; 2 Department of Neurology with Experimental Neurology and Center for Stroke Research Berlin, Charité - Universitätsmedizin Berlin, Berlin, Germany; 3 Department for Neurology, Section of Vascular Neurology, Universitätsklinikum Bonn, Bonn, Germany; 4 Department of Cardiology, Universitätsklinikum Bonn, Bonn, Germany; 5 Department of Neurology, University Hospital Erlangen, Erlangen, Germany; 6 Medizinische Klinik 2, University Hospital Erlangen, Erlangen, Germany; 7 Department of Neurology, Medical Campus Lake Constance, Friedrichshafen, Germany; 8 Department of Cardiology and Intensive Care, Medical Campus Lake Constance, Friedrichshafen, Germany; 9 Department of Neurology, University Medicine Greifswald, Greifswald, Germany; 10 Department of Neurology, University Medical Center Hamburg-Eppendorf, Hamburg, Germany; 11 Department of Neurology, University Hospital Heidelberg, Heidelberg, Germany; 12 Department of Neurology, Leipzig University Hospital, Leipzig, Germany; 13 Department of Cardiology, Leipzig University Hospital, Leipzig, Germany; 14 Neurovascular Center, Department of Neurology, University Medical Center Schleswig-Holstein, Campus Lübeck, Lübeck, Germany; 15 Department of Neurology, Mannheim Center for Translational Neuroscience, Medical Faculty Mannheim, Mannheim, Germany; 16 Department of Internal Medicine and Cardiology, Heart Center Dresden, Medical Faculty and University Hospital Carl Gustav Carus, TUD Dresden University of Technology, Dresden, Germany; 17 Department of Neurology, LMU University Hospital, Munich, Germany; 18 Department of Cardiology, LMU University Hospital, Munich, Germany; 19 Department of Neurology, Klinikum Rechts der Isar, School of Medicine and Health, Technical University of Munich, Munich, Germany; 20 Department of Internal Medicine I, TUM University Hospital, Technical University of Munich, Munich, Germany; 21 Department of Neurology, Klinikum Osnabrück GmbH, Osnabrück, Germany; 22 Department of Neurology and Stroke, Eberhard-Karls University of Tübingen, Tübingen, Germany; 23 Department of Cardiology, Goethe University Frankfurt, Frankfurt, Germany; 24 Department of Cardiology, Angiology and Intensive Care Medicine, Deutsches Herzzentrum Charité, Charité - Universitätsmedizin Berlin, Berlin, Germany; 25 Klinik und Hochschulambulanz für Neurologie, Charite - Universitatsmedizin Berlin, Berlin, Germany; 26 Center for Stroke Research Berlin, Berlin, Germany; 27 German Center for Neurodegenerative Diseases (DZNE), partner site Berlin, Berlin, Germany; 28 German Centre for Cardiovascular Research (DZHK), partner site Berlin, Berlin, Germany; 29 German Center for Mental Health (DZPG), partner site Berlin, Berlin, Germany

**Keywords:** Acute Coronary Syndrome, Percutaneous Coronary Intervention, Pharmacology, Clinical, Stroke, Atherosclerosis

## Abstract

**Background:**

In patients with acute ischaemic stroke (AIS) and concomitant non-ST-elevation acute coronary syndrome (NSTE-ACS), the role of intravenous thrombolysis (IVT) before percutaneous coronary intervention (PCI) is unclear.

**Methods:**

We performed a subanalysis of the PRAISE (PRediction of Acute coronary syndrome in acute Ischemic StrokE) study, a multicentre, prospective observational study in 247 patients with AIS and elevated high-sensitivity cardiac troponin who underwent coronary angiography based on European Society of Cardiology guidelines. The impact of IVT prior to PCI on coronary artery flow (Thrombolysis in Myocardial Infarction (TIMI) score) and myocardial perfusion (TIMI myocardial perfusion score) was compared using Fisher’s exact test and logistic regression analysis, adjusting for time from stroke onset to PCI.

**Results:**

Among 71 patients with AIS undergoing PCI, those who received IVT prior to PCI for NSTE-ACS (33 women; median age 77 (66–82 IQR)) achieved a TIMI grade 3 flow more frequently than those undergoing direct PCI (97% vs 79%; p=0.04). Regression analysis indicated a trend toward improved coronary artery flow with IVT (adjusted OR 8.5, 95% CI 0.9 to 75.3; p=0.05). Myocardial perfusion did not differ between groups (p=0.06).

**Conclusions:**

This subanalysis suggests that IVT before PCI may enhance coronary artery flow in selected patients with NSTE-ACS with AIS. The results of this exploratory subanalysis warrant further investigation, particularly in patients with delayed access to PCI.

**WHAT IS ALREADY KNOWN ON THIS TOPIC**

- Percutaneous coronary intervention (PCI) is the standard treatment for non-ST-elevation acute coronary syndrome, whereas intravenous thrombolysis (IVT), a standard treatment for ischaemic stroke, is not recommended in these patients.

**WHAT THIS STUDY ADDS**

- IVT prior to PCI may favourably modulate the restoration of coronary artery flow in patients with non-ST-elevation acute coronary syndrome and acute ischaemic stroke.

**HOW THIS STUDY MIGHT AFFECT RESEARCH, PRACTICE OR POLICY**

- This analysis provides a basis for research into the potential role of bridging IVT before PCI.

## Introduction

 Percutaneous coronary intervention (PCI) is the standard treatment for ST-elevation myocardial infarction (STEMI) and non-ST-elevation acute coronary syndrome (NSTE-ACS). Rapid intravenous thrombolysis (IVT) with tissue plasminogen activator is recommended only for patients diagnosed with STEMI within 12 hours of symptom onset if PCI is not an immediate option.^1^ In contrast, IVT is the standard of care for patients with acute ischaemic stroke (AIS). IVT provides an additive, beneficial effect on functional outcome when given prior to endovascular thrombectomy.^2 3^

In the PRAISE study (PRediction of Acute coronary syndrome in acute Ischemic StrokE; NCT 03609385) of patients with AIS and elevated high-sensitivity cardiac troponin (hs-cTn) levels meeting the European Society of Cardiology (ESC) ‘rule-in’ criteria for suspected myocardial infarction (MI), dynamic changes in hs-cTn in AIS survivors did not identify type 1 or type 2 MI.^4^ Instead, very high levels of hs-cTn detected type 1 MI with moderate power. In this multicentre, prospective, observational study, 247 patients with AIS who also met ESC guideline rule-in criteria for suspected NSTE-ACS underwent coronary angiography.^1^ An independent endpoint adjudication committee (EAC) diagnosed MI in 126 (51%) of the patients enrolled, including 50 (20%) with type 1 MI and 76 (31%) with type 2 MI. A total of 73 suspected culprit lesions were treated with PCI. Of these, 29 (40%) had received IVT with alteplase for AIS prior to PCI. The PRAISE cohort thus provides a unique patient population that allows for the address of the unanswered question of whether IVT before PCI is associated with a higher degree of epicardial coronary flow after intervention compared with direct PCI.

## Methods

### Study design

This was a retrospective analysis of the PRAISE study population. The trial design and results have been published.^4 5^ Data quality and internal validity were facilitated by external monitoring, central reading by core laboratories, EAC, data safety and monitoring board, critical events committee, prespecified endpoints and sample size calculation.

### Reperfusion endpoints

The central core laboratory blinded to clinical information assessed epicardial coronary artery flow using the Thrombolysis in MI (TIMI) flow score, and myocardial perfusion using the TIMI myocardial perfusion (TMP) score.^6^ In this subanalysis, epicardial coronary artery flow and myocardial perfusion after PCI were compared between patients undergoing PCI with and without prior IVT.

### Statistical analysis

For analysis, TIMI and TMP scores were each dichotomised as 3 vs 0–2, with a TIMI score of 3 indicating complete filling of the distal coronary bed and a TMP score of 3 indicating complete myocardial perfusion. Groups of patients undergoing PCI with and without prior IVT were compared using Fisher’s exact test. Logistic regression analysis was performed to assess the effect of IVT on outcomes adjusted for time from stroke onset to PCI. A p value of <0.05 was considered statistically significant. Analysis was performed with SPSS (IBM SPSS Statistics for Windows, V.29.0.2.0 Armonk, New York, USA).

## Results

### Patients

**Figure 1:**
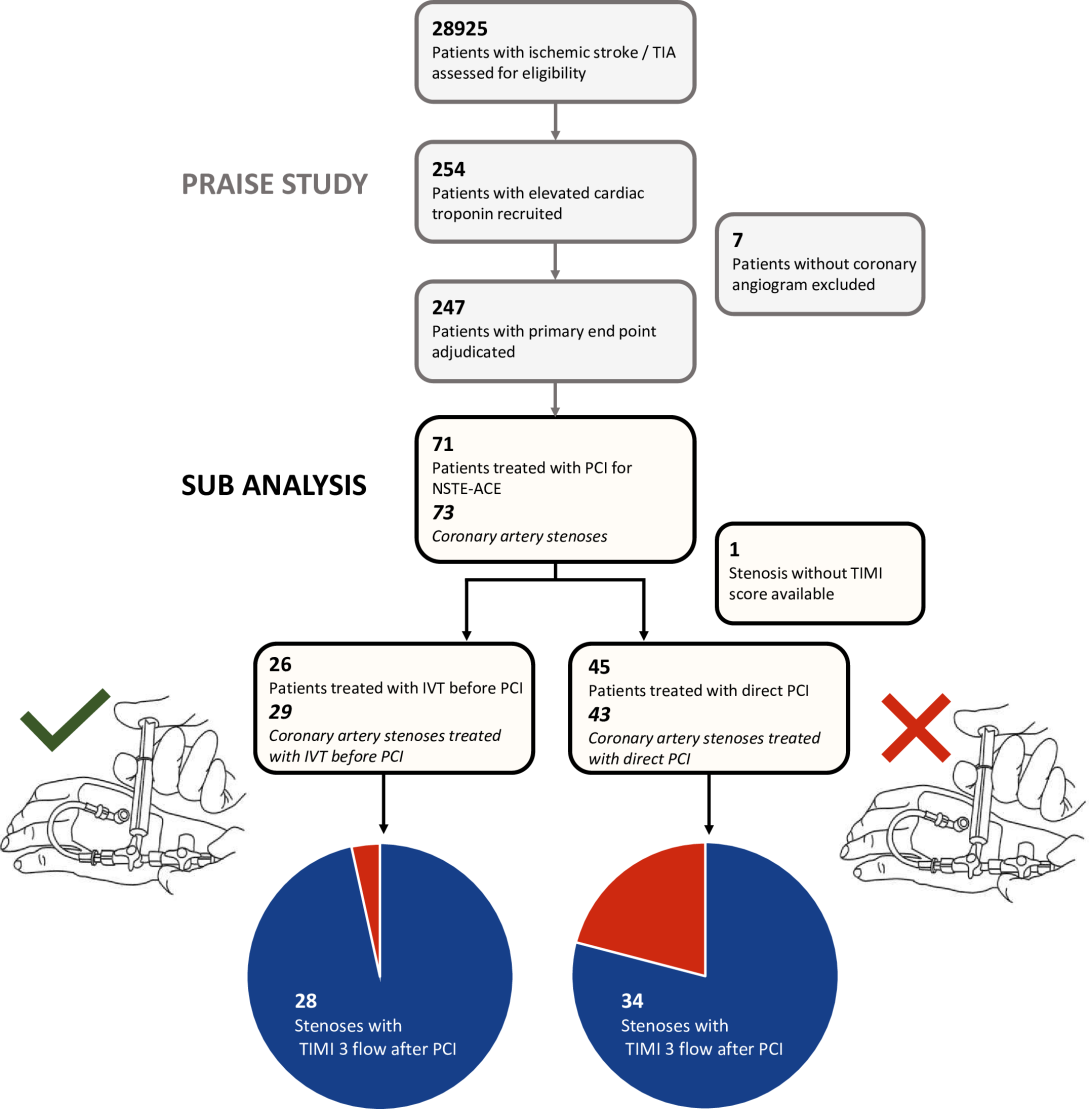
Study flowchart. Study flowchart illustrating the screening and inclusion of patients in the PRAISE main study^3^ (upper chart with grey boxes) as well the selection process and inclusion of patients and corresponding coronary artery stenoses in the subgroup analysis (lower chart with black boxes). IVT, intravenous thrombolysis; NSTE-ACS, non-ST elevation-acute coronary syndrome; PCI, percutaneous coronary intervention; PRAISE, PRediction of Acute coronary syndrome in acute Ischemic StrokE; TIA, transient ischaemic attack; TIMI, Thrombolysis In Myocardial Infarction.

We included 71 patients with NSTE-ACS who underwent PCI (33 women; median age 77 (66–82 IQR)), resulting in 72 stenoses with available TIMI score out of a total of 73 stenoses confirmed by the central imaging core laboratory in this analysis. The study design is shown in figure 1. Population characteristics are shown in table 1.

**Table 1:** Baseline characteristics of patients undergoing PCI for NSTE-ACS with and without prior IVT

	PCI all (N=71)	PCI without IVT (n=45)	PCI with IVT (n=26)	P value
Age, median (IQR)	77 (66–82)	79 (67–83)	74 (65–81)	0.171
Age ≥75 years, n (%)	39 (55)	29 (64)	10 (39)	0.034
Sex (female), n (%)	33 (47)	21 (47)	12 (46)	0.967
Symptom-onset-to-angiography (d), median (IQR)	2 (1–3)	3 (1–4)	2 (0–3)	0.096
Diabetes mellitus, n (%)	24 (34)	18 (40)	6 (23)	0.146
Hypertension, n (%)	61 (86)	40 (89)	21 (81)	0.343
Dyslipidaemia, n (%)	31 (44)	17 (38)	14 (54)	0.221
Known CAD, n (%)	20 (28)	15 (33)	5 (19)	0.203
Atrial fibrillation, n (%)	19 (27)	13 (29)	6 (23)	0.594
Heart failure, n (%)	3 (4)	1 (2)	2 (8)	0.550
Previous stroke, n (%)	17 (24)	10 (22)	7 (27)	0.655
People who currently smoke, n (%)	18 (25)	9 (20)	9 (36)	0.170
People who quit smoking, n (%)	16 (23)	9 (20)	7 (28)	0.168
Intravenous thrombolysis (alteplase), n (%)	26 (37)	0 (0)	26 (100)	n.a.
Thrombectomy, n (%)	9 (13)	5 (11)	4 (15)	0.716
Heart rate (BPM), median (IQR)	77 (65–87)	77 (66–89)	77 (62–85)	0.500
Systolic blood pressure (mm Hg), median (IQR)	139 (122–150)	140 (131–160)	129 (119–146)	0.034
Diastolic blood pressure (mm Hg), median (IQR)	78 (70–89)	78 (72–89)	78 (67–89)	0.814
Beta blocker, n (%)	38 (54)	24 (53)	14 (54)	0.955
Statin, n (%)	51 (72)	31 (69)	20 (77)	0.557
NIHSS at study entry*, median (IQR)	2 (0–5)	2 (0–5)	2 (1–7)	0.152
GRACE score, median (IQR)	144 (121–159)	142 (120–155)	146 (123–167)	0.470
Serum creatinine (mg/dL), median (IQR)	1.0 (0.8–1.2)	1.0 (0.8–1.2)	1.0 (0.8–1.2)	0.990
Haemoglobin (g/L), median (IQR)	134 (116–149)	133 (118–147)	138 (116–150)	0.508
Culprit lesion present, n (%)	35 (49)	19 (42)	16 (62)	0.138
ACS-typical ECG changes, n (%); (n=53)	23 (43)	12 (33)	11 (65)	0.031
LVEF (%), median (IQR); (n=58)	56 (51–61)	57 (52–61)	54 (39–59)	0.130
Wall motion abnormality, n (%); (n=59)	15 (25)	7 (19)	8 (35)	0.187
Dynamic hs-cTn change >50%, n (%)	26 (37)	15 (33)	11 (42)	0.450
Dynamic hs-cTn change >20%, n (%)	38 (54)	22 (49)	16 (62)	0.303
hs-cTn on admission (multiples of ULN), median (IQR)	6.4 (3.2–17.6)	6.6 (3.0–19.6)	5.9 (3.4–11.3)	0.500

Baseline characteristics of the study population of the PRAISE subanalysis including patients undergoing PCI for NSTE-ACS with and without prior IVT.

* Symptom-onset-to-angiography refers to coronary angiography.

† In the PRAISE study, the NIHSS score was assessed at the time of enrolment and no later than 72 hours after admission.

ACS, acute coronary syndrome; BPM, beats per minute; CAD, coronary artery disease; GRACE score, Global Registry of Acute Coronary Events score; hs-cTn, high-sensitivity cardiac troponin; IVT, intravenous thrombolysis; LVEF, left ventricular ejection fraction; n.a., not assessed; NIHSS, National Institutes of Health Stroke Scale; NSTE-ACS, non-ST-elevation acute coronary syndrome; PCI, percutaneous coronary intervention; PRAISE, PRediction of Acute coronary syndrome in acute Ischemic StrokE; ULN, upper limit of normal.

### Coronary artery flow

TIMI grade 3 flow was achieved more frequently in coronary stenoses treated with IVT before PCI than in those treated with direct PCI (n=28 (97%) vs n=34 (79%); p=0.04). In regression analysis, IVT before PCI was associated with an 8.5-fold higher odds of achieving TIMI grade 3 flow; however, this association just failed to reach statistical significance (aOR=8.5, 95% CI (0.9 to 75.3); p=0.05).

### Myocardial perfusion

The TMP score was available for 70 stenoses. Although TMP grade 3 perfusion was numerically four times more common after IVT (n=2 (5%) vs n=6 (21%)), the difference did not reach statistical significance (p=0.06). After adjustment, IVT was not associated with reduced microvascular perfusion (aOR=4.9, 95% CI (0.9 to 26.6); p=0.07).

### Safety endpoints

In this subgroup of the PRAISE cohort, there were no cases of intracranial haemorrhage or major extracranial bleeding with causal relationship to coronary angiography or PCI. Among patients who underwent stent implantation, three serious adverse events with adjudicated causal relationship to coronary angiography/PCI occurred in two patients: one patient (treated with IVT) suffered a recurrent AIS, and another patient (not treated with IVT) developed an in-stent thrombosis, leading to recurrent MI and death.

## Discussion

Our analysis suggests that IVT before PCI for NSTE-ACS may favourably modulate the restoration of epicardial coronary artery flow in patients with AIS. This observation warrants discussion and future studies, as data on the use of IVT in this indication are lacking and the current ESC guideline does not address this issue.^1^

In the American Heart Association guideline, IVT is explicitly not recommended for patients with NSTE-ACS due to a lack of efficacy and potential harm, independent of concomitant stroke.^7^ The authors emphasised the need for further research on the use of fibrinolytics in ACS patient subgroups with bundle branch block, ST-depression and other ECG abnormalities besides ST-segment elevation, as the available data on these groups was limited and the observed effects of IVT differed between them. For instance, patients with ACS without ST-segment elevation but with bundle branch block exhibited a tendency toward reduced mortality after IVT. Therefore, the authors could not rule out a moderate beneficial effect of IVT in selected patients with NSTE-ACS. However, this was before the era of PCI, which has largely made IVT for myocardial ischaemic events obsolete. Nevertheless, further research on the use of IVT prior to PCI could indeed be beneficial, because pretreatment with fibrinolytics could enhance early coronary reperfusion, especially when PCI is delayed or unavailable.^8^

Furthermore, improvements in fibrinolytic agents and adjunctive therapies could mitigate previous risks and potentially improve outcomes when combined with contemporary PCI techniques. The T-TIME (A Trial of Low-dose Adjunctive alTeplase During prIMary PCI) study showed that low-dose periprocedural alteplase had no effect on the extent of microvascular obstruction as measured by contrast-enhanced cardiac MRI in patients with STE-ACS undergoing PCI.^9^ However, alteplase was manually infused during angiography before stent implantation, so it remains unclear whether earlier systemic administration of the lytic agent might have had a greater effect on epicardial coronary artery flow due to a time-dependent mechanism. There are no confirmatory trials on the concept of bridging IVT before PCI. Nevertheless, for patients with NSTE-ACS who do not undergo early invasive angiography within 24 hours of diagnosis, there is a longer time window for pharmacological treatment, during which the potential effect of IVT on restoring epicardial coronary artery flow could be explored in future research.

Our observation was derived from a prospective multicentre cohort study with blinded adjudication of outcomes by a core laboratory, ensuring high internal validity. However, this unplanned, post hoc analysis of a small subset of the cohort is subject to methodological limitations. The lack of prespecification increases the risk of type 1 error, while unmeasured confounders may distort the observed associations. In addition, selection bias may reduce the external validity of the findings. In this subgroup of the PRAISE cohort, serious adverse events were limited to patients who underwent stent implantation. However, given the small sample size of our subanalysis, this observation is limited in its generalisability and must be interpreted with caution. Taken together, the results of our analysis cannot provide conclusive evidence of a treatment effect due to lack of statistical power and the risk of equivocal associations. Rather, they are hypothesis-generating and may provide a basis for investigating potential time-dependent treatment effects of IVT before PCI in NSTE-ACS. This research should evaluate efficacy and safety outcomes and may include the use of tenecteplase for IVT, which recently demonstrated superior efficacy compared with alteplase in AIS.^10^

## Supplementary material

10.1136/openhrt-2025-003567 online supplemental file 1

## Data Availability

Data are available upon reasonable request.
