# Supplementary material for: Intravenous thrombolysis before percutaneous coronary intervention in patients with non-ST-elevation acute coronary syndrome and acute ischaemic stroke: a subanalysis of the PRAISE study
Source: Open Heart. 2025 Sep 29;12(2):e003567. doi: 10.1136/openhrt-2025-003567 (PMC12481274; doi:10.1136/openhrt-2025-003567)

A Subanalysis of the

PRAISE

Study

Intravenous Thrombolysis before Percutaneous Coronary Intervention in Patients with Non-ST-Elevation Acute Coronary Syndrome and Acute Ischaemic Stroke

Among 71 stroke patients undergoing PCI, those who received IV thrombolysis prior to PCI for NSTE-ACS achieved a TIMI grade 3 flow more frequently than those undergoing direct PCI
(97% vs. 79%; P=0.04).

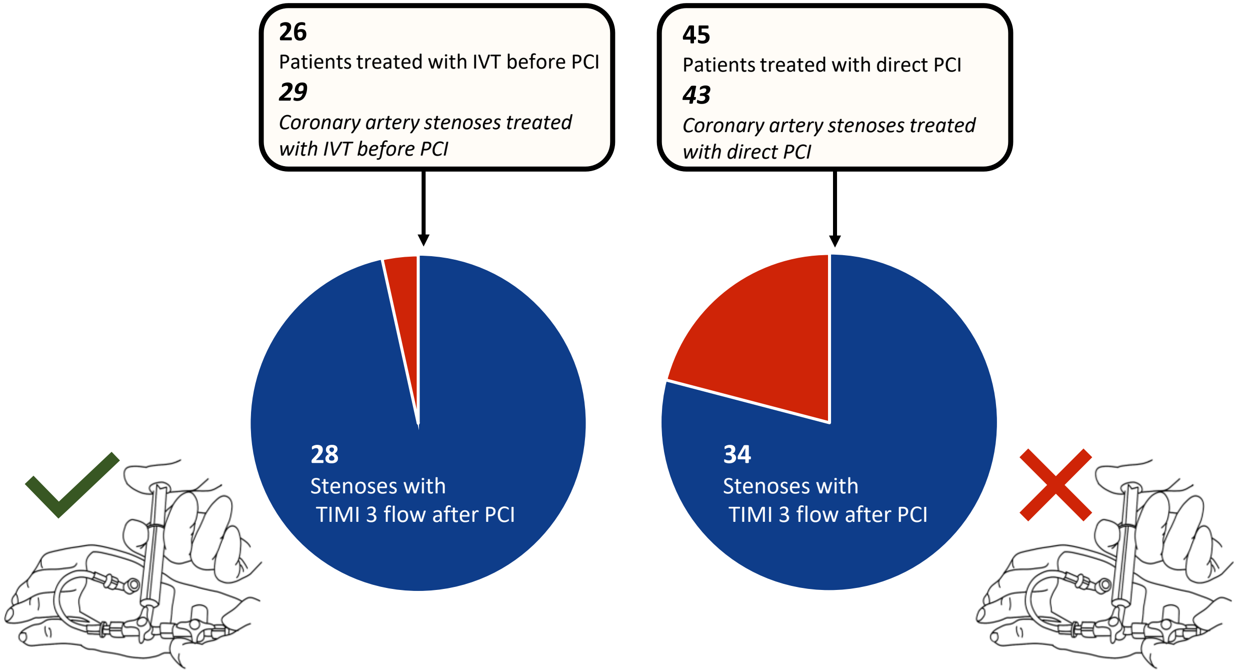

Supplement: online supplemental file 1 [file openhrt-12-2-s001.pdf]
